# ^18^F-FDG and ^18^F-NaF PET/CT Global Assessment of Large Joint Inflammation and Bone Turnover in Rheumatoid Arthritis

**DOI:** 10.3390/diagnostics13132149

**Published:** 2023-06-23

**Authors:** Natasha Reddy, William Y. Raynor, Thomas J. Werner, Joshua F. Baker, Abass Alavi, Mona-Elisabeth Revheim

**Affiliations:** 1 Department of Radiology, Hospital of the University of Pennsylvania, 3400 Spruce Street, Philadelphia, PA 19104, USA; nr522@drexel.edu (N.R.); william.raynor@pennmedicine.upenn.edu (W.Y.R.); tom.werner@pennmedicine.upenn.edu (T.J.W.); abass.alavi@pennmedicine.upenn.edu (A.A.); 2 Division of Rheumatology, University of Pennsylvania, 3400 Spruce Street, Philadelphia, PA 19104, USA; joshua.baker@pennmedicine.upenn.edu; 3 Division of Rheumatology, Corporal Michael J. Crescenz VA Medical Center, 3900 Woodland Avenue, Philadelphia, PA 19104, USA; 4 Department of Epidemiology and Biostatistics, University of Pennsylvania, 423 Guardian Drive, Philadelphia, PA 19104, USA; 5 The Intervention Center, Division of Technology and Innovation, Oslo University Hospital, Sognsvannsveien 20, 0372 Oslo, Norway; 6 Division of Radiology and Nuclear Medicine, Oslo University Hospital, Sognsvannsveien 20, 0372 Oslo, Norway; 7 Institute of Clinical Medicine, Faculty of Medicine, University of Oslo, Problemveien 7, 0315 Oslo, Norway

**Keywords:** FDG, NaF, fluoride, PET, CT, MRI, knee, hip, sacroiliac, joint, rheumatoid arthritis, osteoarthritis, psoriatic arthritis, ankylosing spondylitis, BMI, body weight

## Abstract

Rheumatoid arthritis (RA) involves chronic inflammation of synovial joints, causing pain, stiffness, and limited mobility. ^18^F-sodium fluoride (NaF) is a PET tracer whose uptake reflects bone turnover, while ^18^F-fludeoxyglucose (FDG) shows glucose metabolism and can serve as a marker for inflammation. The aim of this study is to determine the feasibility of calculating the FDG and NaF mean standardized uptake value (SUVmean) in the knee joint, hip joint, and sacroiliac (SI) joint of RA patients and to determine their association with patient characteristics. Prospective FDG-PET/CT as well as NaF-PET/CT imaging was performed on 18 RA patients. The global SUVmean was calculated on FDG-PET/CT and NaF-PET/CT images using a semiautomated CT-based method of segmentation. FDG and NaF uptake were found to be significantly correlated in the knee (r = 0.77, *p* < 0.001), but not in the hip and SI joints. In the knee, both NaF SUVmean and FDG SUVmean were significantly correlated with body weight, BMI, leptin, and sclerostin levels (*p* < 0.05). NaF SUVmean was significantly positively correlated with BMI and leptin for both the hip and SI joints (*p* < 0.05). No significant correlation was observed between either PET parameter and age, height, erythrocyte sedimentation rate (ESR), and interleukins 1 and 6 (IL-1 and IL-6); however, FDG was correlated with inflammatory markers such as C-reactive protein (CRP) and patient global visual analogue scale (VAS-PtGlobal) in some joints. In this study, both FDG and NaF uptake were quantified in large joints of patients with RA using CT segmentation. NaF and FDG SUVmean were correlated with clinical variables related to body weight and adiposity, suggesting that degenerative joint disease may play a larger role in influencing the uptake of these tracers in large joints than RA disease activity. FDG and its correlation with markers of inflammation such as CRP and VAS-PtGlobal suggests that this tracer may serve as a more specific marker for RA disease activity than NaF. Larger prospective and longitudinal data are necessary to gain a better understanding of the roles of FDG and NaF in evaluating RA joint activity in these joints.

## 1. Background

Rheumatoid arthritis (RA) is a chronic autoimmune disorder that targets joint tissue, resulting in inflammation, pain, and stiffness. Autoimmune attack of the synovial membrane lining the joints causes swelling that damages the cartilage and ligaments, leading to pain and instability. Upper extremities are often affected; however, destruction of weight-bearing joints of the lower extremities is associated with decreased function in activities of daily living [1]. For example, the knee joint is affected in up to 30% of RA patients [2], and knee disorders are a major cause of morbidity in the elderly [3]. Early arthritis and synovitis of hip joints is less common but is often overlooked and may be subclinical in nature. Approximately 15% of RA patients had impaired hip joint function within one year of disease onset, and this progressed to 28% after 5 years [4].

In order to evaluate RA involvement in joints, various imaging modalities can be performed, such as magnetic resonance imaging (MRI), ultrasonography, computerized tomography (CT), and plain radiographs. Conventional bone scans are often used for musculoskeletal disorders, but these images have lower resolution and are therefore not as effective for detecting uptake. Positron emission tomography (PET) using ^18^F-fluorodeoxyglucose (FDG) is highly sensitive for early detection of inflammatory processes [3]. ^18^F-sodium fluoride (NaF) is a PET tracer that conveys bone turnover. Reactive bone formation in the context of degenerative joint disease results in increased NaF uptake [5]. As a molecular imaging modality, PET may provide evidence for pathological molecular changes before structural changes are evident on MRI, CT, or conventional radiography [3]. Early diagnosis and treatment is crucial for treating various forms of arthritis, and it is thus important to evaluate which methods are the most sensitive indicators of inflammation and bone turnover.

While an abundance of previous research has focused on imaging with MRI, considering it the “gold standard”, recent studies have been analyzing the usefulness of PET in combination with CT. In fact, when compared to MRI, FDG PET showed promising results in many of the RA studies in both the more commonly studied small joints and the less often studied large joints [6,7,8,9,10,11,12,13,14,15,16]. The aim of this study is to explore the feasibility of calculating the FDG and NaF mean SUV (SUVmean) in the knee joint, hip joint, and sacroiliac (SI) joint of RA patients and to determine the association of these values with clinical characteristics of patients with the disease.

## 2. Methods

### 2.1. Patient Population

Eighteen RA patients who met the 2010 American College of Rheumatology classification were recruited in a prospective study conducted at the Corporal Michael J. Crescenz Veterans Affairs Medical Center in Philadelphia, PA. Exclusion criteria for this study included the presence of metabolic bone disease, active malignancy, or CT imaging within the past 6 months. FDG- and NaF-PET/CT imaging was performed in all subjects. Eighteen subjects were chosen since this will provide sufficient power to detect correlation coefficients (r) of 0.62 or greater with a power of 0.8 and alpha of 0.05. We have considered that correlations below this magnitude would be less likely to be clinically relevant or reproducible in larger studies and clinical trials.

The patient population included 5 females and 13 males whose ages ranged from 25 to 69, with an average age (SD) of 57.3 (11.9) years. The duration of their RA diagnosis was an average of 11.91 years, ranging from 0.13 to 36 years. All of the patients were also on RA medications, which generally included methotrexate, prednisone, sulfasalazine, and/or adalimumab.

All work was performed in compliance with the Health Insurance Portability and Accountability Act (HIPAA) and approval was obtained from the Philadelphia VA Medical Center Internal Review Board (IRB # 01427).

### 2.2. Clinical Variables

Variables that were applied in this study include descriptive factors such as age and height, as well measures of body fat such as BMI, leptin, sclerostin, and total fat. Indicators of RA and inflammation such as C-reactive protein (CRP), erythrocyte sedimentation rate (ESR), interleukin-1 (IL-1), and interleukin-6 (IL-6) were also evaluated. All laboratory data represent serum values obtained within 2 weeks of PET/CT imaging. DAS28-ESR and DAS28-CRP, which represent Disease Activity Scores in 28 joints based on clinical and lab data, were also applied [17]. Visual analog scale-based patient global assessment (VAS-PtGlobal) was determined by a handwritten mark made by the patient along a 10 cm line, which represented a continuum between “no pain” and “worst pain”. Patient characteristics are presented in Table 1.

### 2.3. Imaging

In order to obtain the PET/CT images for this study, the patients were asked to follow specific instructions for each tracer. First, FDG imaging was conducted, before which they were required to fast for 6 h. Fingerstick glucose readings were performed beforehand to ensure a reasonable range for accurate imaging and all patients had a glucose concentration confirmed to be below 8 mmol/L. An IV catheter was used to administer 0.11 mCi/kg radiolabeled FDG before imaging. NaF imaging was conducted during the second visit and no fasting or fingerstick glucose measurements were required. An IV catheter was used to administer 0.08 mCi/kg radiolabeled NaF before imaging.

All PET/CT examinations were performed on a Biograph 64 Hybrid PET/CT Imaging System (Siemens Medical Solutions, Inc. Malvern, PA, USA) at the Philadelphia VA Medical Center. The whole-body static FDG PET/CT examination was performed 180 min after administration of FDG. The acquisition time was 3.5 min/bed. The NaF-PET/CT examinations were performed 90 min following tracer injection and the acquisition time was 2.5 min/bed. Corrections were performed for scatter, random coincidences, and scanner dead time, while low-dose CT was used for attenuation correction and anatomic localization.

### 2.4. Image Analysis

In choosing a region of interest for each joint, the goal was to capture parts of the bone that might be altered by the disease process and that fall within a certain distance of the joint. Because subcortical bone is involved in the pathogenesis of RA, our regions of interest captured this area using methods from another study in our lab that was successfully able to capture subcortical bone in order to quantify sodium fluoride uptake in the knees [19].

**Figure 1:**
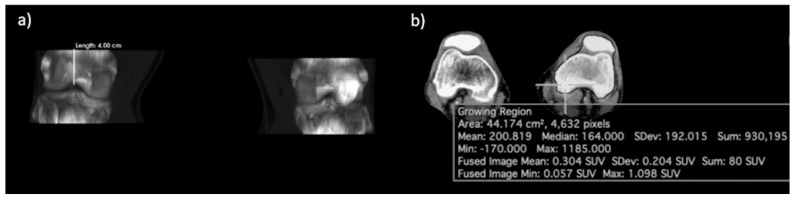
Delineation of the region of interest (ROI) of the knee joint. (**a**) Coronal view of the CT segmentation showing the boundary 4 cm above and below the intercondylar eminence. (**b**) Axial view showing the process of morphological closing.

Regions of interest (ROIs) were defined using a semiautomated method of CT segmentation after anatomical boundaries were manually delineated (Osirix MD Software; Pixmeo, SARL, Bernex, Switzerland). For the knee joint, the superior boundary was defined as the axial plane 4 cm superior to the intercondylar eminence, while the inferior boundary was 4 cm inferior to the intercondylar eminence. Cortical bone of the femur, tibia, and patella was segmented using a growing region algorithm with a lower threshold of 150 HU and upper threshold of 1500 HU. Morphological closing with an element radius of 20 was then applied to obtain the final ROI. The global SUVmean, defined as the average SUV of all voxels included within the ROI, was calculated separately for each knee joint (Figure 1).

**Figure 2:**
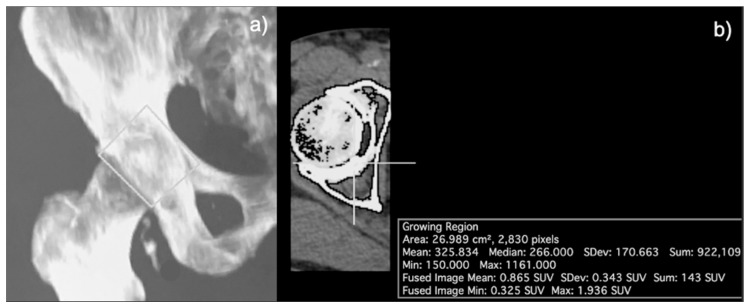
Region of interest (ROI) of the hip joint. (**a**) Coronal view of the CT segmentation showing the rectangular region around the head of the femur and its articulation with the acetabulum, excluding the femoral neck. (**b**) Axial view depicting the morphological closing process.

For the hip joint, the anatomical boundaries were defined as a rectangular region surrounding the entire head of the femur and its articulation with the acetabulum, excluding the femoral neck. More specifically, the lateral border of the rectangular region was defined as the distinction between the femoral neck and femoral head, the medial border was 2 mm lateral to the pelvic brim, and the superior and inferior borders were 1 cm superior and inferior to the edge of the femoral neck, respectively. Again, a lower threshold of 150 HU and upper threshold of 1500 HU was applied to the CT component of the coregistered PET/CT image, followed by morphological closing with an element radius of 20 to segment the skeletal structures within the anatomically defined region. The SUVmean was calculated separately for left and right hips on FDG-PET/CT and NaF-PET/CT images (Figure 2).

**Figure 3:**
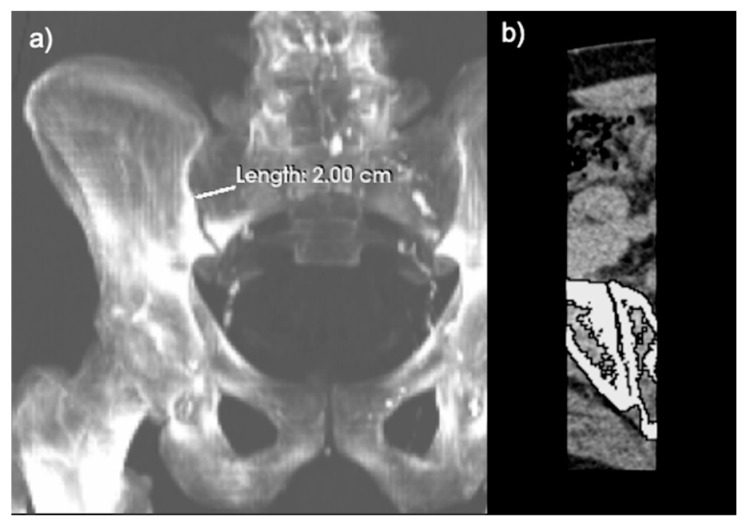
Delineation of the region of interest (ROI) of the sacroiliac (SI) joint. (**a**) Coronal view of the CT segmentation showing the lateral and medial borders forming 2 cm from the articulation of the sacrum and ilium. (**b**) Axial view depicting the process of morphological closing.

The anatomical boundaries of the SI joint were defined as a rectangular region with the lateral and medial borders forming 2 cm laterally and medially from the articulation of the sacrum and the ilium. The superior border and inferior border were defined as the superior and inferior ends of the articulation, respectively. Once again, CT segmentation with a lower threshold of 150 HU and an upper threshold of 1500 HU, followed by morphological closing, was performed on the fused PET/CT image. The SUVmean was calculated separately for left and right SI joints on FDG-PET/CT and NaF-PET/CT images (Figure 3).

### 2.5. Statistical Analysis

For all three joints, paired *t*-tests were used to determine differences in uptake between left and right sides. Differences in laterality have been seen in osteoarthritis, specifically the dominant side being more affected by the disease [20]. Laterality differences have also been seen in the hands of RA patients [21], but fewer have compared laterality in large joints as we investigate here. Univariate linear regressions and Pearson correlations were used to compare PET parameters (averaged over both left and right) with patient characteristics. Scatter plots were used to evaluate the relationships between continuous variables.

## 3. Results

**Figure 4:**
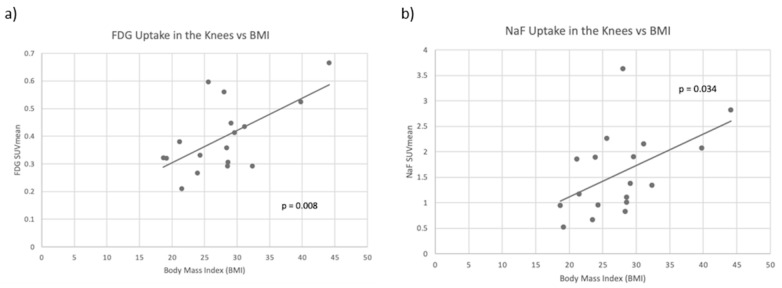
(**a**) Scatter plot of averaged left and right knee FDG SUVmean compared to body mass index (BMI) with a line of best fit. FDG uptake and BMI were significantly positively correlated (*p* = 0.008). (**b**) Scatter plot of averaged left and right NaF SUVmean compared to BMI with a line of best fit. NaF uptake and BMI were significantly positively correlated (*p* = 0.034).

A total of 18 patients, 5 female and 13 male, were included in this study. Patient characteristics are given in Table 1. In the knee joint, FDG and NaF uptake were found to be significantly correlated with each other (r = 0.77, *p* < 0.001). For FDG, the average of the SUVmean was 0.402, while mean NaF uptake in the knee joint was 1.587. Neither NaF SUVmean nor FDG SUVmean showed significant differences in laterality between the knees. This was true even when considering only right-handed subjects. When compared with clinical variables, FDG SUVmean in the knee was significantly correlated with body weight, BMI, and leptin levels, and NaF SUVmean in the knee was significantly correlated with BMI, leptin, and sclerostin. At the knee, neither of the PET parameters were significantly correlated with age, height, or traditional markers of RA disease activity such as sedimentation rate (ESR), C-reactive protein (CRP), Disease Activity Score in 28 joints (DAS28-CRP), IL-1, and IL-6. Scatter plots of averaged FDG SUVmean and NaF SUVmean compared to BMI are shown in Figure 4. All correlation coefficients and *p*-values between clinical variables and tracer uptake in the knee joint are presented in Table 2.

**Figure 5:**
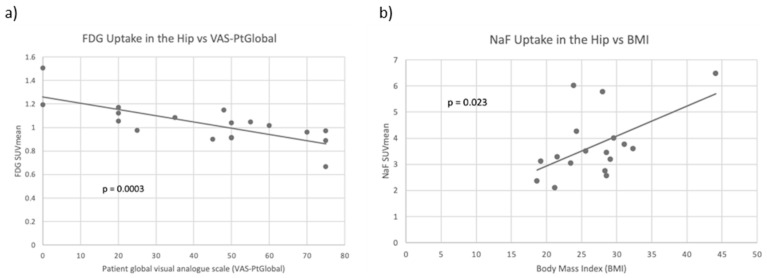
(**a**) Scatter plot of averaged left and right FDG SUVmean compared to patient global visual analogue scale (VAS-PtGlobal) with a line of best fit. FDG uptake and VAS-PtGlobal were significantly negatively correlated (*p* < 0.001). (**b**) Scatter plot of averaged left and right NaF SUVmean compared to BMI with a line of best fit. NaF uptake and body mass index (BMI) were significantly positively correlated in the hip (*p* = 0.023).

In the hip joint, FDG and NaF uptake were not significantly correlated with each other. For FDG, the average SUVmean was 1.03, while the average SUVmean of NaF was 3.73. FDG SUVmean was observed to be higher in the right hip compared to the left hip (*p* < 0.001). No significant differences between right and left hip were observed in the NaF SUVmean. When compared with clinical variables, FDG SUVmean was significantly inversely correlated with DAS28-CRP and with VAS-PtGlobal (*p* = 0.024 and *p* = 0.0003, respectively). VAS-PtGlobal uses a 100 mm Visual Analog Scale (VAS) to represent the patient’s subjective assessment of disease severity. NaF SUVmean at the hip was significantly positively correlated with BMI, leptin, sclerostin, and total fat (*p* < 0.05) but not with age, height, or traditional markers of RA disease activity. Scatter plots of averaged FDG SUVmean compared to VAS-PtGlobal and averaged NaF SUVmean compared to BMI are shown in Figure 5. Correlation coefficients and *p*-values between clinical variables and tracer uptake in the hip joint are presented in Table 3.

**Figure 6:**
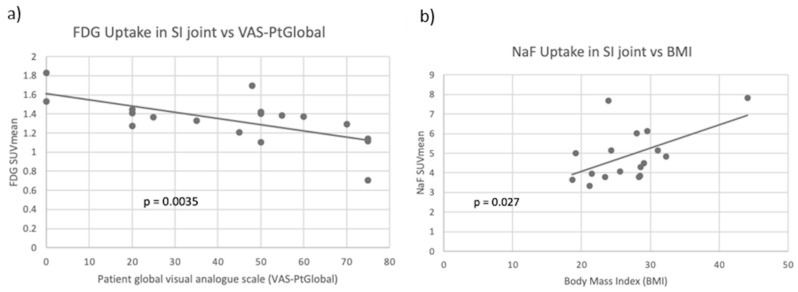
(**a**) Scatter plot of averaged left and right FDG SUVmean compared to patient global assessment of disease activity (VAS-PtGlobal) with a line of best fit. FDG uptake and VAS-PtGlobal were significantly negatively correlated (*p* = 0.004). (**b**) Scatter plot of averaged left and right NaF SUVmean compared to body mass index (BMI) with a line of best fit. NaF uptake and BMI were significantly positively correlated in the SI joint (*p* = 0.027).

In the SI joint, no significant correlation was found between the uptake of FDG and the uptake of NaF. For FDG, the average SUVmean was 1.33, while the average SUVmean for NaF was 4.88. Both the NaF SUVmean and the FDG SUVmean showed significantly higher uptake in the right SI joint compared to the left SI joint (*p* < 0.001). FDG SUVmean was significantly inversely correlated with VAS-PtGlobal (*p* = 0.003). NaF SUVmean was significantly positively correlated with BMI, leptin, and sclerostin (*p* < 0.05). At the SI joint, neither PET parameter had significant associations with age or height, and NaF SUVmean did not correlate with traditional indicators of RA disease activity. Scatter plots of averaged FDG SUVmean compared to VAS-PtGlobal and averaged NaF SUVmean compared to BMI are shown in Figure 6. Table 4 shows all correlation coefficients and *p*-values between clinical variables and tracer uptake in the SI joint.

## 4. Discussion

In our study, we found the quantification of FDG and NaF uptake in large joints in RA patients to be feasible using CT segmentation. Uptake of NaF in the knee, hip, and SI joints was not significantly associated with markers of inflammation but was highly associated with clinical variables representing BMI. FDG uptake in these joints was inversely associated with clinical disease activity. Previous studies using FDG PET [22] and NaF PET [23,24] have shown encouraging results in the detection and evaluation of RA. Detecting and treating RA during its earlier stages is necessary for preventing the development of joint erosions and may affect disease outcomes, even to a remission state [25]. Without early treatment, inflammation will lead to articular damage, especially during the first two years of disease onset, thus requiring more intensive therapy [25]. While RA often causes morbidity in the more well-studied small joints of the hands and feet, it also can result in significant disease burden in larger joints such as the knees and hips [1,2,3,4,5]. We believe there is a need to investigate large joints for subclinical disease and that PET/CT may be an effective way of doing so through quantification of inflammation and bone turnover [13,14,15,16,19,22,23,24].

In the knee joint, neither PET parameter was associated with traditional markers of systemic inflammation such as ESR, CRP, DAS28-CRP, IL-1, and IL-6. Rather, both NaF SUVmean and FDG SUVmean were correlated to clinical variables related to body weight and adiposity. Because the knee is the largest weight-bearing joint in the body, it is reasonable that the knee joint had more inflammation and bone turnover in patients with a higher BMI. A previous study by Al-Zaghal et al. looked at the correlation between FDG and NaF uptake in the knee and BMI [3]. They found that uptake of both tracers in the knee joint was positively correlated with BMI, reinforcing our findings. These results suggest that the uptake seen here is more likely to represent inflammation and bone formation related to degenerative joint disease rather than direct RA activity.

In contrast to the knee joint, uptake of NaF and FDG in the hip joint was found to be associated with clinical variables relevant to RA joint disease. In particular, FDG SUVmean was inversely correlated with VAS-PtGlobal and DAS28-CRP, while no significant correlation with either variable was found with NaF SUVmean. Significant positive correlations were observed between NaF SUVmean and indicators of body fat such as BMI and leptin. Because DAS28-CRP includes markers of inflammation, it is reasonable that they are associated with FDG uptake but not NaF uptake, which better represents bone turnover. Other studies investigating the hip joint did not look at the correlation between uptake and these markers of inflammation, but one large study did compare FDG and NaF uptake with BMI and age [26]. They had similar findings that support ours, as both FDG uptake and NaF uptake in the hip increased with BMI and neither tracer was correlated with age. This further substantiates the connection between increased BMI and greater inflammation and bone turnover.

Since the involvement of the SI joint in RA is controversial, it has rarely been discussed in previous studies. However, it is interesting to compare its tracer uptake with the other large joints proven to be involved in RA. The SI joint had similar results to the hip joint in that uptake of NaF was associated with BMI and leptin and that no significant associations were observed between NaF SUVmean and traditional indicators of RA disease activity. Additionally, FDG SUVmean was inversely correlated with VAS-PtGlobal. The similarity between these two joints may be explained by their combined role in absorbing the impact of walking and other weight-bearing activities, leading to a similar degenerative pattern. As VAS-PtGlobal is a subjective measure of the patient’s assessment of their own disease, the possible involvement of bias could also account for the relationship between VAS-PtGlobal and the hip and SI joint, but not the knee joint.

In the SI joint, both FDG and NaF SUVmean were significantly higher on the right SI joint when compared with the left. Similarly, in the hip joint, FDG SUVmean was observed to be significantly higher on the right side. However, no lateral bias in the uptake of either tracer was observed in the knee joint. One explanation for this is that the majority of right-handed people are also right leg dominant, stepping their right foot forward more often and thus possibly irritating the joints on this side. According to previous studies, RA presentation can vary, appearing symmetrically or asymmetrically depending on the patient, the joint, and the timeline of the disease. One study found that as the number of joints involved in RA increased, the probability of symmetry also increased [27]. Another study found that asymmetry was often seen in small joints but that there was a shift towards symmetry over time [28]. While the SI and hip joints showed laterality, there was no apparent difference in the right and left knee, suggesting that the differences in laterality seen in the hand and other joints may not be seen in the knee. Evidently, more research is warranted regarding laterality in RA.

However, we observed consistency throughout all three joints in terms of NaF SUVmean showing a significant association with indicators of body fat such as BMI, leptin, and sclerostin. In the knee joint, this correlation was also seen with FDG SUVmean. The joints investigated in this study are all large joints in the lower extremities and have a major weight-bearing role. The results indicate that bone turnover in these weight-bearing joints has a close positive relationship with BMI, suggesting that patients with a higher BMI likely require increased turnover in order to support the joint degeneration that is occurring. Because NaF SUVmean was consistently associated with BMI-related variables rather than with indicators of RA disease activity, degenerative joint disease may play a larger role in influencing NaF uptake than RA disease activity.

BMI and obesity-related variables were also a recurring theme in several previous studies which connected BMI to disorders such as osteoarthritis (OA), psoriatic arthritis (PA), ankylosing spondylitis (AS), and, of course, RA. One study found that adiponectin and leptin levels were higher in RA than in controls and that adiponectin was involved in bone destruction and damage [29]. Although we did not find a correlation with adiponectin, NaF SUVmean was significantly correlated with leptin in all three joints and FDG SUVmean was correlated with leptin in the knee. An OA study concluded that each 1 kg loss in overweight in obese adults was associated with a four-fold decrease in force and load on the knee, which was observed to a clinically meaningful level [30]. Another RA study concluded that BMI appears to be causally associated with an increased risk of RA [31]. Because our own study and many others [29,30,31,32,33,34,35] support the correlation between BMI and RA, the possibility that weight loss may decrease disease burden warrants further investigation, especially in combination with early detection using FDG- and NaF-PET/CT.

While NaF SUVmean was consistently associated with BMI, FDG SUVmean was more often associated with indicators of RA disease activity such as DAS28-CRP (in the hip) and VAS-PtGlobal (in the hip and SI joint). This is consistent with previous studies, where FDG-PET/CT was able to quantify inflammation-mediated bone damage [6] and FDG uptake seemed to be a better measure of RA disease activity than NaF SUVmean [3]. NaF PET/CT has not been used often to study RA but has been the focus of many OA and ankylosing spondylitis studies. This is reasonable because OA and AS mainly affect bone and NaF uptake represents bone metabolism, whereas RA involves more inflammation, which is probably better studied using FDG. A study using both NaF and FDG tracers showed that NaF uptake was mostly located in bone while FDG was found in surrounding tissue, further substantiating this idea [36].

Neither of the PET parameters showed significant correlations with age or sex in any of the joints that were studied. This was surprising since bone turnover and inflammation vary with age and sex, such as in post-menopausal women. A possible explanation for the absence of correlation with age and sex could be the smaller sample size of our study, which varied widely in regard to age, ranging from 25 to 69. The bulk of the patients were male, while less than a third of them were female. However, one larger study with 116 subjects whose ages ranged from 21 to 75 had similar results to us, in which they also did not identify a correlation between age and NaF or FDG uptake [26]. A study that did find a correlation with age and FDG uptake, but not NaF uptake, looked at the knee joints of 97 healthy patients [3]. They found that FDG uptake increased with age in the soft tissue compartment but not the osseous compartment. Since our study focused only on the osseous compartment, this could account for the variability. Age may affect the level of inflammation more in the surrounding tissue of the joint than in the actual osseous compartment. Further investigations involving both the bone and the surrounding tissue in healthy patients and patients with RA may be of value.

While some studies utilized similar methods to ours [3,19,26], others followed varying procedures that may explain any differences in results. One study assessing inflammation and bone formation in the knee joint measured SUVmean for FDG- and NaF-PET/CT, delineating the ROI as 4 cm above and below the midpoint between lateral and medial intercondylar tubercles, just as our study did [3]. Another study using FDG- and NaF-PET/CT measured SUVmean in the hip joint, delineating the ROI as a rectangular region including only the femoral head, acetabulum, and articular cartilage, which is identical to our own study [26]. Both of these studies used a larger sample size and had similar results to ours, supporting the hypothesis that increased BMI is correlated to increased reactive bone formation and inflammation.

Some other studies, however, used ROIs manually drawn by nuclear physicians and radiologists [8,11,37,38] or created through the use of different types of software and dedicated workstations [9,22,39,40]. Many studies also used SUVmax [12,40,41] instead of SUVmean, which could have varied results. Additionally, while some other papers used control groups, many did not. These differences should be considered when comparing results and when designing future studies. Differences in sample size, the presence or absence of control groups, different patient populations, varying disease severities, comorbid conditions, and anti-inflammatory medications also need to be considered when comparing results, as well as in future studies.

A limitation in this study is the smaller sample size, so future prospective studies including larger prospective and longitudinal data are warranted. Few studies have looked at RA in large joints, and even fewer have used FDG- and NaF-PET/CT to do so due to the relatively new application of this technique to such diseases. Evaluation of a larger number of joints in RA patients using FDG- and NaF-PET/CT may also provide further clarity regarding the utility of these imaging methods and help compare the varying involvement of different joints in this disease. The various treatment regimens of the patients in this study, which included drugs such as methotrexate and adalimumab, may have also influenced the relationships between radiotracer uptake and markers of disease activity. Future longitudinal studies which assess FDG and NaF activity both before and after initiating therapy are essential for a better understanding of treatment-related changes.

In conclusion, this study focused on using novel imaging methods to quantify NaF and FDG uptake in the large joints of patients with RA. The association between FDG and NaF uptake in these joints and BMI, as well as other indicators of excess adiposity, suggests that comorbid OA is also a key underlying contributor to the abnormalities observed in these joints. These observations suggest that NaF- and FDG-PET/CT imaging methods may be a way to compare and distinguish OA and inflammatory disease in various contexts.

## Figures and Tables

**Table 1:** Characteristics of the patient population including age, weight, height, BMI, RA duration, inflammatory markers (CRP, ESR, IL-1, and IL-6), DAS28-ESR, DAS28-CRP, and VAS-PtGlobal.

Characteristic	*n* (%)
Total	18
Male	13 (72.2)
Female	5 (27.8)
Left-handed	4 (22.2)
Right-handed	13 (72.2)
Ambidextrous	1 (5.6)
Age (years)	
Median (IQR)	60.5 (14.3)
Range	25–69
Weight (kg)	
Median (IQR)	77.7 (14.5)
Range	47.0–130.3
Height (cm)	
Median (IQR)	170.8 (18.8)
Range	153.1–188.7
BMI (kg/m^2^)	
Median (IQR)	28.5 (7.2)
Range	18.7–44.1
RA duration (years)	
Median (IQR)	8.4 (14.8)
Range	0.1–35.8
ESR (mm/h)	
Median (IQR)	21 (32)
Range	3–66
CRP (mg/dL)	
Median (IQR)	1.2 (1.0)
Range	0.5–3.1
IL-1 (pg/mL) (normal, 0–5 pg/mL) [18]	
Median (IQR)	0.3 (0.1)
Range	0.2–0.9
IL-6 pg/mL (normal, 5–15 pg/mL) [18]	
Median (IQR)	2.3 (2.3)
Range	0.5–8.7
DAS28-CRP	
Median (IQR)	4.1 (2.4)
Range	1.6–5.4
VAS-PtGlobal	
Median (IQR)	46.5 (26.0)
Range	4–75

IQR, interquartile range; BMI, body mass index; CRP, C-reactive protein; ESR, erythrocyte sedimentation rate; IL-1, interleukin-1; IL-6, interleukin-6; DAS28, Disease Activity Score in 28 joints; VAS-PtGlobal, visual analog scale-based patient global assessment of disease activity.

**Table 2:** Correlation coefficients and *p*-values between clinical variables and tracer uptake in the knee joint. Both NaF uptake and FDG uptake in the knee were significantly correlated with body mass index (BMI), leptin, and total fat, while FDG was also correlated with weight and NaF was inversely correlated with sclerotin.

Knee Joint	FDG SUVmean		NaF SUVmean	
	R-Value	*p*-Value	R-Value	*p*-Value
Age	0.362	0.153	−0.039	0.877
Weight	0.506	0.038	0.457	0.056
Height	−0.193	0.458	0.017	0.947
BMI	0.619	0.008	0.501	0.034
Leptin	0.500	0.008	0.509	0.031
Sclerostin	−0.545	0.444	−0.489	0.040
Total Fat	0.520	0.001	0.541	0.031
CRP	0.304	0.133	0.261	0.296
ESR	−0.124	0.961	0.246	0.341
IL-1	0.356	0.161	0.182	0.470
IL-6	0.307	0.231	0.138	0.585
DAS28-CRP	−0.034	0.850	−0.008	0.976
VAS-PtGlobal	−0.152	0.773	−0.231	0.357

**Table 3:** Correlation coefficients and *p*-values between clinical variables and tracer uptake in the hip joint. NaF uptake was positively correlated with body mass index (BMI), leptin, and total fat and inversely correlated with sclerostin. FDG uptake was inversely correlated with DAS28-CRP and VAS-PtGlobal.

Hip Joint	FDG SUVmean		NaF SUVmean	
	R-Value	*p*-Value	R-Value	*p*-Value
Age	0.066	0.796	−0.381	0.131
Weight	−0.051	0.840	0.412	0.101
Height	−0.001	0.998	−0.256	0.322
BMI	−0.096	0.703	0.548	0.023
Leptin	−0.038	0.122	0.61	0.009
Sclerostin	0.077	0.762	−0.583	0.014
Total Fat	−0.120	0.635	0.504	0.039
CRP	0.318	0.198	0.368	0.147
ESR	0.340	0.181	0.091	0.739
IL-1	0.337	0.172	−0.026	0.922
IL-6	0.299	0.229	−0.094	0.721
DAS28-CRP	−0.529	0.024	0.083	0.752
VAS-PtGlobal	−0.755	<0.001	−0.096	0.713

**Table 4:** Correlation coefficients and *p*-values between clinical variables and tracer uptake in the SI joint. NaF uptake correlated positively with body mass index (BMI) and leptin and inversely with sclerostin. FDG uptake correlated inversely with VAS-PtGlobal.

SI Joint	FDG SUVmean		NaF SUVmean	
	R-Value	*p*-Value	R-Value	*p*-Value
Age	−0.055	0.827	−0.423	0.091
Weight	0.087	0.732	0.379	0.134
Height	0.302	0.224	−0.295	0.250
BMI	−0.111	0.661	0.536	0.027
Leptin	−0.442	0.066	0.592	0.012
Sclerostin	−0.038	0.882	−0.506	0.038
Total Fat	−0.125	0.623	0.462	0.062
CRP	0.325	0.189	0.231	0.372
ESR	0.215	0.407	0.113	0.677
IL-1	0.248	0.321	−0.170	0.515
IL-6	0.335	0.175	−0.190	0.465
DAS28-CRP	−0.459	0.055	0.249	0.336
VAS-PtGlobal	−0.650	0.003	0.113	0.666

## Data Availability

The datasets used and/or analyzed during the current study are available from the corresponding author on reasonable request.
